# Diabetes Mellitus and Its Treatment

**Published:** 1899-03

**Authors:** 


**Diabetes Mellitus and its Treatment.** By R. T. WILLIAMSON, M.D. Pp. xi., 417. London: Young J. Pentland. 1898.

It is rarely that we meet with a work in which an author not only gives a clear account of valuable observations and studies of his own, but also a full exposition of the theories and experiments of other investigators, especially when these relate to minor branches of the subject where the writer's own interest is less keen. Dr. Williamson, however, has carried his thoroughness not only into his own extensive researches at first hand, and has not been satisfied with producing a brilliant essay on the subject from his own point of view, but he has presented us with probably the best existing abstract of the numerous papers on every aspect of the subject which have appeared during the last twelve years. It was a task much needed, for the recent literature has been so voluminous and scattered that reference to it had become practically impossible; and, apart from the scientific advances which have been made, a large number of practical improvements have been recorded both in treatment, diagnosis, and chemical tests.

It is refreshing to read the new light thrown on the usual tests, and of the errors usually committed. No precaution seems too small to notice, but we find that the author relies chiefly (even in detecting very small amounts of sugar) on Fehling's solution, phenylhydrazin, and fermentation. In difficult cases they are all used successively in the above order, and Fehling's test again applied to the urine which has gone through the fermentation process. The over-sensitiveness of phenylhydrazin is avoided by boiling for two minutes only, a valuable improvement which gets rid of the minute crystals formed from normal urine by the older method. Dr. Williamson confesses that the question of traces of sugar in healthy urine is still unsolved, though he shows reasons for disbelieving Pavy's estimate of .05 per cent. In any case, these and other ordinary tests are unaffected by it.

After discussing some of Pavy's recent controversies, with a brevity for which the reader will be grateful, he gives us a summary of the recent work relating to the pancreas, and finds reason to think that in certain cases diabetes is directly due to pancreatic disease, though it is not possible to say with certainty during life whether it is due to this or other causes.

It is important to find that he has rarely failed to find the tubercle bacillus in the phthisis of diabetes, though he recognises that rare cases occur where it is absent.

The analysis of the symptoms of coma is especially interesting, and not least the presence of a yellowish deposit in the urine, consisting of enormous numbers of granular casts, which is very often found just before the onset of an attack. Here also the author's test from the blood reaction with methylene blue becomes invaluable for diagnosis if no urine can be obtained.

The chapter on foods and treatment is particularly full and careful. The gain of weight and general condition of the patient is put forward as of more importance than a mere decrease of sugar, desirable as this is. When we come to the question of bread substitutes, the author shows the little value of many of the usual preparations, for which, indeed, a reduced quantity of ordinary bread can be given with more comfort and equally good results. He recommends a cheap and palatable preparation made from aleuronat and cocoanut meal, and having a very low percentage of starch, for use when a rigid diet is indicated. The receipts for the manufacture of this and other articles are clear and practical; and if there is little fresh to be learnt from the chapter on drugs, we feel that the subject is not one on which much can be said at present.

The book is issued in Mr. Pentland's best style, the printing and references are clear and good, and the volume will be of value to every one who is studying the theory of the disease or has to treat it.

